# Epistasis and the sensitivity of phenotypic screens for beta thalassaemia

**DOI:** 10.1111/bjh.13241

**Published:** 2014-12-17

**Authors:** Bridget S Penman, Sunetra Gupta, David J Weatherall

**Affiliations:** 1 Department of Zoology, University of OxfordOxford, UK; 2 Weatherall Institute of Molecular Medicine, Oxford University, John Radcliffe HospitalOxford, UK

**Keywords:** thalassaemia, haemoglobinopathies, genetic disorders, epistasis, screening programmes

## Abstract

Genetic disorders of haemoglobin, particularly the sickle cell diseases and the alpha and beta thalassaemias, are the commonest inherited disorders worldwide. The majority of affected births occur in low-income and lower-middle income countries. Screening programmes are a vital tool to counter these haemoglobinopathies by: (i) identifying individual carriers and allowing them to make informed reproductive choices, and (ii) generating population level gene-frequency estimates, to help ensure the optimal allocation of public health resources. For both of these functions it is vital that the screen performed is suitably sensitive. One popular first-stage screening option to detect carriers of beta thalassaemia in low-income countries is the One Tube Osmotic Fragility Test (OTOFT). Here we introduce a population genetic framework within which to quantify the likely sensitivity and specificity of the OTOFT in different epidemiological contexts. We demonstrate that interactions between the carrier states for beta thalassaemia and alpha thalassaemia, glucose-6-phosphate dehydrogenase deficiency and Southeast Asian Ovalocytosis have the potential to reduce the sensitivity of OTOFTs for beta thalassaemia heterozygosity to below 70%. Our results therefore caution against the widespread application of OTOFTs in regions where these erythrocyte variants co-occur.

Severe forms of alpha and beta thalassaemia have been estimated to affect approximately 68000 births annually (Modell & Darlison, 2008; Weatherall, 2010). Individuals who carry thalassaemic genes are protected against death from malaria infection (Haldane, 1949; Willcox *et al*, 1983; Flint *et al*, 1986; Mockenhaupt *et al*, 2004; Williams *et al*, 2005; Wambua *et al*, 2006; Taylor *et al*, 2012); the highest frequencies of thalassaemias are thus found in regions which were historically malarious (Table I).

**Table I:** A selection of the highest reported frequencies of alpha and beta thalassaemia

	Methods used	Highest frequencies observed
Alpha thalassaemia	Exact gene frequencies determined by DNA analysis	Up to 0·17 in Pakistan (Khan *et al*, 2003) 0·41 on the coast of Kenya (Wambua *et al*, 2006) Up to 0·68 in Oceania (Flint *et al*, 1986) Up to 0·83 in the Tharu population of the Terai region of Nepal (Modiano *et al*, 1991)
	Alpha thalassaemia trait determined phenotypically	Up to 0·37 in Sardinian populations (Cao *et al*, 2008)
Beta thalassaemia	Carrier frequencies determined phenotypically	Up to 0·15 in Sardinian villages (Siniscalco *et al*, 1966) Up to 0·11 in Liberia, specifically in members of the Kru tribal group (Willcox, 1975) Up to 0·09 in Gujerat (Patel *et al*, 2012) 0·11 in Maewo, Melanasia (Bowden *et al*, 1987)

Adult haemoglobin (HbA) is a tetramer made up of two alpha and two beta globin chains. The thalassaemias arise when production of either alpha or beta globin is compromised (Weatherall & Clegg, 2001). The vast majority of alpha thalassaemia is caused by whole gene deletions. There are two alpha globin genes in tandem on chromosome 16 (*HBA1* and *HBA2*), and it is possible for one or both to be deleted, leading to four main alpha thalassaemic states. The loss of a single alpha globin (αα/-α) is phenotypically silent; the loss of two (-α/-α or αα /--) causes a mild anaemia; the loss of 3 (-α/--) causes a variably severe anaemia called Haemoglobin H disease and the loss of 4 (--/--) is usually fatal *in utero*. Beta thalassaemia can be caused by many different mutations that reduce or eliminate expression of the beta globin gene (*HBB*) on chromosome 11. Throughout this paper we shall use the symbol ‘β^T^’ to represent a generic beta thalassaemic mutation. Inheriting a gene for beta thalassaemia from both parents (β^T^β^T^) usually leads to a life-threatening, transfusion-dependent anaemia (Cooley anaemia, or thalassaemia major), whilst heterozygosity (the carrier state, ββ^T^) causes only a mild anaemia.

Many of the characteristics of the thalassaemias derive not only from a limited supply of either alpha or beta globin, but from the accumulation of unpaired globin chains of the subunit produced at the normal rate (Weatherall & Clegg, 2001). If both alpha and beta thalassaemia are inherited together, there may be an amelioration of symptoms due to a balancing out of the rate of chain synthesis. This effect can explain the surprisingly mild course of the disease in some beta thalassaemia homozygotes (Kan & Nathan, 1970; Weatherall *et al*, 1981; Wainscoat *et al*, 1983), and it has been noted that such epistasis can make it difficult to detect carriers of severe thalassaemic mutations (Bowden *et al*, 1987; Weatherall & Clegg, 2001; Law *et al*, 2003).

Any programme to identify carriers of beta thalassaemia involves an initial survey to identify individuals with unusual red blood cell indices, followed by further analysis of abnormal samples. Table II summarizes some key phenotypic characteristics of alpha and beta thalassaemic individuals. In a screen for beta thalassaemia heterozygotes, microcytosis or low cellular haemoglobin levels are typical phenotypic traits used to distinguish abnormal samples in the first instance. The presence of elevated levels of Haemoglobin A_2_ (HbA_2_) in those samples is the gold standard used to identify definite carriers of beta thalassaemia in the second stage of the screen. Nevertheless, there do exist ‘silent’ beta thalassaemic mutations with normal HbA_2_ levels (Weatherall & Clegg, 2001) and there can be problems interpreting samples with borderline elevated HbA_2_, so even the gold standard does not necessarily represent 100% accuracy.

**Table II:** The phenotypic signatures of thalassaemia traits

	Alpha thalassaemia: αα/−α	Alpha thalassaemia: −α/−α	Heterozygous beta thalassaemia: ββ^T^
Microcytosis: MCV <80 fl	43% of sample had MCV <80 fl (*n* = 191)	100% of sample had MCV <80 fl (*n* = 77)	100% of males had MCV <80 fl 100% of females had MCV <80 fl (Total *n* = 83)
Hypochromia: <27 pg Hb per cell	64% of sample had MCH <27 pg (*n* = 184)	100% of sample had MCH <27 pg (*n* = 75)	100% of males had MCH <27 pg 100% of females had MCH <27 pg (Total *n* = 83)
High RBC count >5·7 × 10^12^/l (males) or >5 × 10^12^/l (females)	31% of males had RBC >5·7 × 10^12^/l (*n* = 77) 41% of females had RBC >5 × 10^12^/l (*n* = 102)	64% of males had RBC >5·7 × 10^12^/l (*n* = 30) 73% of females had RBC >5 × 10^12^/l (*n* = 45)	43% of males had RBC >5·7 × 10^12^/l 58% of females had RBC >5 × 10^12^/l (Total *n* = 83)
Elevated HbA_2:_ >3·4%	HbA_2_ levels indistinguishable from controls with full complement of HBA1 and HBA2 genes (Maude *et al*, 1985)	HbA_2_ levels indistinguishable from controls with full complement of HBA1 and HBA2 genes (Maude *et al*, 1985)	100% of sample had HbA_2_ >3·4% (*n* = 83)

Weatherall and Clegg (2001) provide a complete review of the thalassaemias and their pathophysiology. In Table II, beta thalassaemia statistics were extrapolated from the means and standard deviations reported by Knox-Macaulay *et al* (1973) in British thalassaemia heterozygotes, assuming each phenotype to be normally distributed within the sample. MCV, MCH and red cell blood count statistics for alpha thalassaemia were extrapolated from Table 11·4 in Weatherall and Clegg (2001) using values reported for 16+ years of age, and similarly assuming normal distributions. HbA_2_ levels in alpha thalassaemic individuals are based on a study of alpha thalassaemic children in Jamaica (Maude *et al*, 1985).

MCV, mean corpuscular volume; MCH, mean corpuscular haemoglobin; Hb, haemoglobin; RBC red blood cell.

The most reliable method to quantify microcytosis is to use an automated cell counter. However, a simpler and cheaper method, used in regions where resources are limited, is to detect cells with low red blood cell indices using the one tube osmotic fragility test or OTOFT (Kattamis *et al*, 1981). The osmotic fragility of a red blood cell is related to its size and shape: the smaller the cell, the more resistant it should be. Similarly, low cellular haemoglobin may also be linked to an increase in osmotic resistance. To perform the OTOFT, a drop of blood is placed in a tube containing buffered NaCl solution (typically 0·36% NaCl) or glycerine-saline; the tube is allowed to stand, then the degree of lysis is assessed photometrically or by visual inspection. In the latter case the test is known as the Naked Eye Single Tube Red cell Osmotic Fragility Test or NESTROFT (Raghavan *et al*, 1991). If lysis is below a certain level, the sample is deemed microcytic and should be referred for further testing.

Figure1A compares reported mean corpuscular volume (MCV) values of red blood cells from individuals carrying different combinations of thalassaemic mutations (Sanna *et al*, 1980; Kanavakis *et al*, 1982; Melis *et al*, 1983; Rosatelli *et al*, 1984; Maccioni & Cao, 1985; Weatherall & Clegg, 2001), together with the MCVs of blood samples that tested either positive or negative in a OTOFT (Yazdani *et al*, 2008). The low MCV of carriers of either alpha or beta thalassaemia can be elevated by the coinheritance of alpha *and* beta thalassaemic mutations, due to the aforementioned ameliorative effect of balancing out globin chain synthesis, such that the range of MCV values for −α/−α ββ^T^ individuals overlaps the range of MCV values that tested negative in the OTOFT. This suggests there may be a risk of −α/−α ββ^T^ individuals being missed by the OTOFT screen.

**Figure 1:**
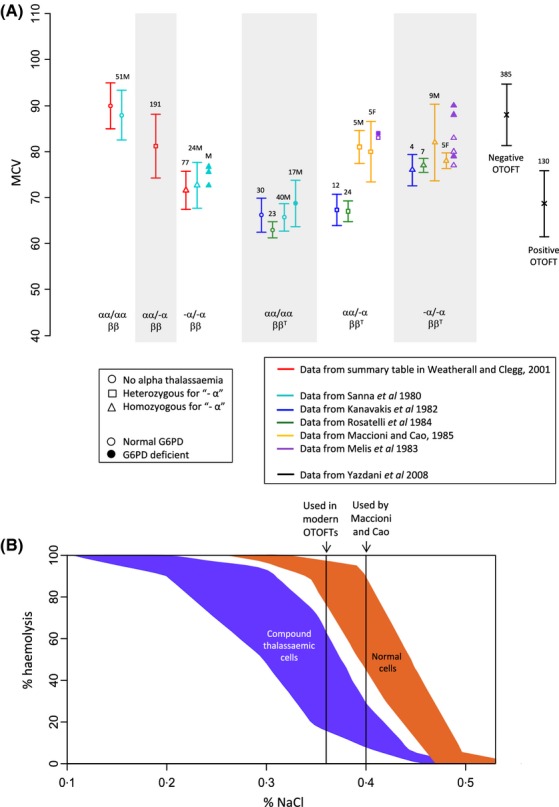
Mean corpuscular volumes (MCVs) and osmotic fragility profiles for different thalassaemic genotypes. In panel (A), bars represent the mean ± 1 standard deviation for the MCV of each indicated genotype; sample sizes are indicated above each bar. Where a sample was stated to consist only of males or females, this has been indicated with ‘M’ or ‘F’. Markers lacking error bars represent reported values from single individuals of the indicated genotype. The ‘normal’ and alpha thalassaemia data are taken from Table 11·4 of Weatherall and Clegg (2001), and are MCV values for individuals >16 years of age. The beta thalassaemic and alpha-beta thalassaemic data are taken from Kanavakis *et al* (1982), Rosatelli *et al* (1984), Maccioni and Cao (1985), Sanna *et al* (1980) and Melis *et al* (1983). Sanna *et al* (1980) reported MCV values for alpha thalassaemic individuals defined phenotypically; we have assumed this sample to represent homozygotes for ‘−α’. The one tube osmotic fragility test (OTOFT) MCV data are from Yazdani *et al* (2008). Panel (B) illustrates haemolysis rates (y axis) for different concentrations of NaCl (x axis) for compound alpha-beta thalassaemia heterozygotes and normal cells (Maccioni & Cao, 1985). No distinction was made in that study between different forms of alpha thalassaemia. The Sanna *et al* data in panel (A) were extracted from graphs in Sanna *et al* (1980) using *GetData Graph Digitizer*. The curves in panel (B) were extracted using *GetData Graph Digitizer* from: *Journal of Medical Genetics*, Maccioni, L. & Cao, A., 22, 374–376, copyright 1985. With permission from BMJ Publishing Group Ltd.

However, the correlation between osmotic fragility and MCV is not perfect. Despite their increased MCV, the osmotic fragility profile of doubly heterozygous alpha-beta thalassaemic cells remains distinct from that of normal cells – as illustrated in Fig1B (Maccioni & Cao, 1985). Osmotic fragility tests may therefore be able to detect properties of thalassaemic cells that are unrelated to MCV. Nevertheless, the osmotic fragility of thalassaemic cells still presents a potential problem for modern one-tube tests. Maccioni and Cao (1985) were able to detect the presence of heterozygous beta thalassaemia, even in the simultaneous presence of alpha thalassaemia, by applying a OTOFT at 0·4% NaCl. As can be seen in Fig1B, this concentration offers the maximum discriminative power between normal and alpha-beta thalasssaemic cell types. However, many modern day OTOFTs are performed at lower NaCl concentrations (Table III). A test performed at 0·36% NaCl may be more prone to missing some −α/αα ββ^T^ or −α/−α ββ^T^ samples.

**Table III:** A selection of field surveys assessing the sensitivity of OTOFTs (including NESTROFT) as a test for beta thalassaemia

Study	Population and location	Sensitivity of OTOFT for beta thalassaemia	Testing solution	Thalassaemia frequency
Sumera *et al* (2012)	Dow Diagnostics Research and Reference Laboratory in Karachi, Pakistan	93%	0·36% NaCl	73/503 subjects had elevated HbA_2_ levels: frequency of β^T^ = 0·07
Mamtani *et al* (2006, 2007)	Sindhi individuals living in Nagpur, India	93·4%, 91%	0·36% NaCl	Jawahirani *et al* (2007) studied the same population, and reported sub-population beta thalassaemia frequency estimates of β^T^ = 0·08–0·2
Chakrabarti *et al (*2012)	Pregnant Rajbanshi women in West Bengal	95%	0·36% NaCl	17/500 subjects had elevated HbA_2_ levels: frequency of β^T^ = 0·017
Manglani *et al* (1997)	Field camps in Gujarat and Maharashtra	94·4%	0·36% NaCl	142/830 subjects had elevated HbA_2_ levels: frequency of β^T^ = 0·085
Wiwanitkit *et al* (2002)	Pregnant Thai women	100%	0·36% NaCl	3/213 had beta thalassaemia trait: frequency of β^T^ = 0·007 1/213 had ‘alpha thalassaemia trait confirmed by genotyping’
Sirichotiyakul *et al* (2004)	Maharaj Nakorn Chiang Mai, 446 singleton pregnancies (2002)	97·6%	0·45% glycerine saline solution	14/446 had elevated HbA_2_: frequency of β^T^ = 0·016 33/446 had positive result for the SEA alpha thalassaemic deletion (a double deletion of alpha globin); frequency of SEA deletion = 0·037
Tongprasert *et al* (2010)	Maharaj Nakorn, Chiang Mai, 477 singleton pregnancies, (2002–2008)	100% (for both beta thalassaemia and heterozygosity for the SEA deletion)	0·45% glycerine saline solution	28/417 had elevated HbA_2_: frequency of β^T^ = 0·034. 33/417 had positive result for the SEA alpha thalassaemic deletion; frequency of SEA deletion = 0·040.
Mehta (2002)	Lohana community	98% sensitivity	0·36% NaCl	49/450 had elevated HbA_2_ (decision to test for HbA_2_ seems to have been partly based on NESTROFT result, so this is perhaps not a true population estimate): frequency of β^T^ = 0·054
Mehta (2002)	Antenatal clinics	100% sensitivity	0·36% NaCl	68/2350 had elevated HbA_2_ (decision to test for HbA_2_ seems to have been partly based on NESTROFT result, so this is perhaps not a true population estimate): frequency of β^T^ = 0·015
El-Beshlawy *et al* (2007)	Siblings of hospital patients (children), families had no history of haematological disease	87% sensitivity	0·36% NaCl	90/1000 had unambiguously elevated HbA_2,_ giving a frequency of β^T^ of 0·045. If borderline elevated HbA_2_ samples are also assumed to carry beta thalassaemia, the frequency of β^T^ in the sample is 0·051

Table III only includes population surveys where nothing was known about the thalassaemia status of participants prior to their recruitment, and where it is possible to assess the likely population frequency of beta thalassaemia from information given in the paper. Other studies have assessed the sensitivity of OTOFTs by specifically targeting individuals already known to have beta thalassaemia (or the families of individuals already known to have thalassaemia major). Such surveys typically report very high sensitivity values (Thomas *et al*, 1996; Bobhate *et al*, 2002) but, as noted by Mamtani *et al* (2006), are likely to overestimate sensitivities by including such a high frequency of samples that are extremely likely to be positive. OTOFT, one tube osmotic fragility test; NESTROFT, naked eye single tube red cell osmotic fragility test.

A further difficulty in detecting individuals carrying both alpha and beta thalassaemia may arise from the variation in another protein: glucose-6-phosphate dehydrogenase (G6PD) (Melis *et al*, 1983). G6PD, encoded by *G6PD* on the X chromosome, helps to protect erythrocytes from oxidative damage. Nucleotide substitutions in *G6PD* can disrupt the function of the enzyme, causing G6PD deficiency. G6PD deficiency tends to increase the MCV of cells from beta thalassaemia heterozygotes, but not to the same extent as alpha thalassaemia. However, individuals with the −α/−α ββ^T^ genotype who are also G6PD-deficient may enjoy a further boost to their MCV: these individuals tended to have the highest MCV values reported by Melis *et al* (1983). G6PD deficiency was also suggested by Maccioni and Cao (1985) as one of the factors that might hide the presence of beta thalassaemia in OTOFT screens, due to its MCV-increasing effects.

Finally, a recent case report from Thailand (Fucharoen *et al*, 2007) reveals yet another complication for osmotic fragility-based screens for carriers of beta thalassaemia. Fucharoen *et al* (2007) described a Thai female heterozygous for both beta thalassaemia and Southeast Asian Ovalocytosis (henceforth SAO, caused by heterozygosity for a deletion in the Band 3 gene *SLC4A1*), whose osmotic fragility profile is entirely normal. It seems that the altered membrane properties of SAO cells counteract the increased osmotic resistance that usually results from heterozygous beta thalassaemia. The authors noted that this masking effect could create a pitfall for osmotic screening in regions where SAO is common (Fucharoen *et al*, 2007).

In this paper, we present an analytical framework to investigate the sensitivity and specificity of OTOFTs as first-stage screens for carriers of beta thalassaemia, in the context of epistasis with alpha thalassaemia, G6PD deficiency and SAO. Our analysis focuses in particular on three unknown factors: (i) the probability of OTOFTs detecting carriers of beta thalassaemia in the simultaneous presence of homozygosity or heterozygosity for a common alpha thalassaemic deletion; (ii) the potential for G6PD deficiency to modify the chances of double heterozygotes for both alpha and beta thalassaemia being detected, and (iii) the probability of heterozygosity for a common alpha thalassaemic deletion causing a false positive OTOFT result.

We demonstrate that high frequencies of alpha thalassaemia combined with G6PD deficiency and SAO have the potential to reduce the sensitivity of OTOFTs to unacceptable levels. Our results underscore the fact that any decision to roll out OTOFT-based beta thalassaemia screening to a new population should take account of that population's pre-existing genetic background. Our framework offers a potential tool for guiding such decisions, but also highlights the need for an improved understanding of the performance of OTOFTs for different genotypes.

## Methods

We considered a population in which alpha thalassaemia is caused by the deletion of a single alpha globin gene (HBA2 or HBA1) i.e. ‘−α’, also designated α^+^; by far the most common type worldwide. Let us represent beta thalassaemic alleles using the symbol β^T^; the band 3 deletion with the symbol σ^DEL^ and G6PD-deficient X chromosomes with the symbol X^DEF^. There are 81 female genotypes in the population, made up of all possible combinations of alpha globin genotype (αα/αα, −α/αα or −α/−α); beta globin genotype (ββ, ββ^T^, β^T^β^T^); SAO genotype (σσ, σσ^DEL^, σ^DEL^σ^DEL^) and G6PD genotype (XX, XX^DEF^*,* X^DEF^X^DEF^). There are, however, only 54 possible male genotypes because males are hemizygous for the X chromosome (XY or X^DEF^Y). We assume that none of the individuals being screened are homozygous for σ^DEL^, because no individuals homozygous for the SAO deletion in *SLC4A1* have been reported to survive. We similarly assume that individuals with beta thalassaemia major (β^T^β^T^) are excluded from thalassaemia screening programmes, since their illness will have become apparent in childhood.

In this analysis, *a*,*b*,*s* and *c* represent the allele frequencies of ‘−α’, β^T,^ σ^DEL^ and X^DEF^ in the population, and *m* represents the proportion of individuals who test positive for microcytosis in a OTOFT. The latter can be split into two quantities: the proportion of individuals who are both microcytic and prove to have elevated HbA_2_ levels at the next phase of the screen (*m*_*+*_), and the proportion of individuals who are microcytic but lack elevated HbA_2_ (*m*_*0*_). Of the *m*_*0*_ individuals, some will carry alpha thalassaemia. These we shall designate *m*_*0*α_.

As noted in the introduction, epistasis between alpha and beta thalassaemia may mean that not all individuals with *both* alpha and beta thalassaemia will necessarily test positive in a OTOFT. To allow for these uncertainties, we introduce the following parameters: *g*_1_, the proportion of non-G6PD-deficient −α/ααββ^T^ σσ samples that test positive in a OTOFT; *g*_2_, the proportion of non-G6PD-deficient −α/−α ββ^T^ σσ samples that test positive in a OTOFT and *g*_*3*_, the proportion of non-G6PD-deficient −α/αα ββ σσ samples that test positive in a OTOFT.

It is also possible that any OTOFT masking effect of alpha thalassaemia on beta thalassaemia detection depends on the simultaneous presence of G6PD deficiency. To allow for this, we shall also introduce parameters *g*_*4:*_ the proportion of −α/ααββ^T^ σσ X^DEF^X^DEF^ females or −α/ααββ^T^ σσ X^DEF^Y males to test positive in a OTOFT, and *g*_*5*:_ the proportion of −α/−αββ^T^ σσ X^DEF^X^DEF^ females or −α/−αββ^T^ σσX^DEF^Y males to test positive in a OTOFT.

We shall assume that the coinheritance of SAO alongside either alpha or beta thalassaemia eliminates the osmotic resistance of each, leading to a negative OTOFT result. There is evidence that this occurs for beta thalassaemia (Fucharoen *et al*, 2007); the osmotic resistance of cells with both alpha thalassaemia and SAO has not been tested.

Finally, thalassaemia is not the only condition that could give rise to microcytosis, and hence positive results in a field microcytosis test. Iron deficiency anaemia (typically caused by poor nutrition, or intestinal parasites) is extremely common in many of the regions where thalassaemia is also found, and can also lead to smaller than usual red blood cells. The parameter *d* represents the proportion of the sample with non-genetic causes of a positive OTOFT.

Eqs 1–4 show how *m*_*+(i)*_; *m*_*0*α_ and *m*_*0*_ are calculated. *m*_*+(i)*_ will be different for males and females, so we present two different equations, using _(1)_ for females and _(2)_ for males. *m*_*+(i)*_ and *m*_*0(i)*_ are equal to the number of true positives and false positives for beta thalassaemia in the sample. Eqs 5–7 give the numbers of false negatives (*n*_*f(i)*_) and true negatives (*n*_*t(i)*_), and finally Eqs 2007 and 1986 calculate the sensitivity and specificity of a OTOFT-like test for beta thalassaemia.


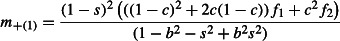
(1)

where

*f*_1_ = 2(1 − *a*)^2^*b*(1 − *b*) + *g*_1_4*a*(1 − *a*)*b*(1 − *b*) + *g*_2_2*a*^2^*b*(1 − *b*)
*f*_2_ = 2(1 − *a*)^2^*b*(1 − *b*) + *g*_4_4*a*(1 − *a*)*b*(1 − *b*) + *g*_5_2*a*^2^*b*(1 − *b*)

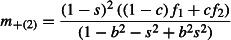
(2)

where *f*_1_ and *f*_2_ are defined as for Eq. 2.


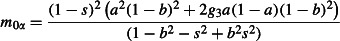
(3)


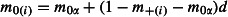
(4)


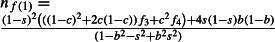
(5)

where

*f*_3_ = (1 − *g*_1_)4*a*(1 − *a*)*b*(1 − *b*) + (1 − *g*_2_)2*a*^2^*b*(1 − *b*)
*f*_4_ = (1 − *g*_4_)4*a*(1 − *a*)*b*(1 − *b*) + (1 − *g*_5_)2*a*^2^*b*(1 − *b*)

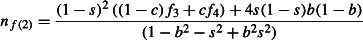
(6)

where *f*_3_ and *f*_4_ are defined as for Eq. 5.


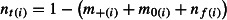
(7)


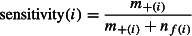
(8)


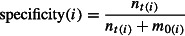
(9)

## Results

### Increasing frequencies of alpha thalassaemia and G6PD can reduce the sensitivity of OTOFTs for beta thalassaemia

Sensitivity measures the reliability with which a negative test result indicates the absence of an underlying condition. Highly sensitive tests are crucial when offering genetic counselling. From the equations given in the Methods, the critical factors determining the sensitivity of OTOFTs are (i) the frequencies of SAO, alpha thalassaemia and G6PD deficiency in the population and (ii) the values of *g*_1_, *g*_2_*, g*_4_ and *g*_5_.

**Figure 2:**
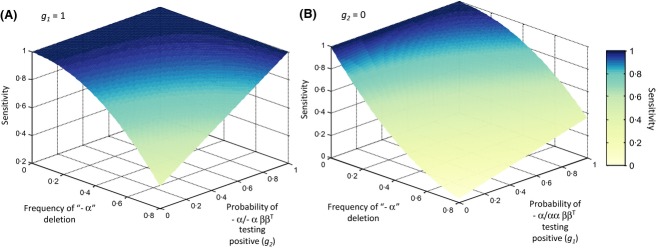
How the sensitivity of one tube osmotic fragility tests for beta thalassaemia may change under the influence of epistasis with alpha thalassaemia. The population frequency of alpha thalassaemia and *g*_2_ and *g*_1_ respectively were varied as indicated in the two surfaces. In surface (A), *g*_*1*_ = 1; in surface (B) *g*_2_* *=* *0. The frequencies of SAO and G6PD deficiency are zero. Other parameters (d, *g*_3_, beta thalassaemia frequency) have no effect on the sensitivity of the test.

We shall first examine the potential impact of alpha thalassaemia alone on OTOFT sensitivity. Whenever it is possible for alpha thalassaemia to mask the presence of heterozygous beta thalassaemia, there is a decline in sensitivity with increasing alpha thalassaemia frequency (Fig2). In the surface described in Fig2A, heterozygosity for a single alpha globin deletion (α−/αα) does not mask the detection of beta thalassaemia carriers by a OTOFT, hence *g*_1_* *=* *1, but homozygosity for a single alpha globin deletion (−α/−α) can. As the masking effect of −α/−α increases there is a striking non-linear decline in sensitivity with increasing alpha thalassaemia frequency. The surface illustrated in Fig2B describes a more extreme situation, where homozygosity for a single alpha globin deletion always masks the detection of heterozygous beta thalassaemia (*g*_2_* *=* *0), and −α/αα can simultaneously have a masking effect. As the masking effect of −α/αα increases, the decline in sensitivity with increasing alpha thalassaemia frequency becomes more linear – and therefore lower frequencies of alpha thalassaemia could potentially have a bigger impact on sensitivity.

However, as we saw in Fig1B, *complete* masking of beta thalassaemia carriers by the simultaneous presence of alpha thalassaemia (i.e. *g*_2_* *=* *0) seems unlikely – there remains a difference in osmotic fragility between doubly heterozygous alpha-beta thalassaemic cells and normal cells. What if, as suggested in the introduction, it is a combination of alpha thalassaemia and G6PD deficiency which has the most powerful masking effect?

**Figure 3:**
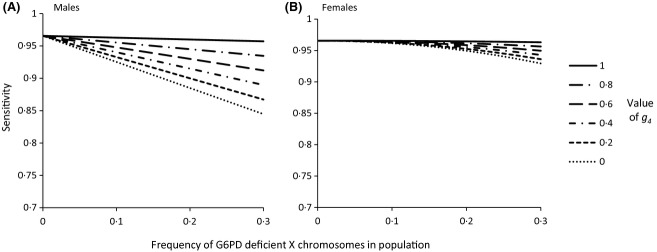
How the sensitivity of one tube osmotic fragility tests for beta thalassaemia may change under the influence of epistasis with alpha thalassaemia and G6PD deficiency. Here *g*_1_* *=* *0·95, *g*_2_* *=* *0·75, *g*_4_ was varied as indicated in the figure, and *g*_5_ = 0. The population frequency of alpha thalassaemia was assumed to be 0·25 and the population frequency of G6PD deficiency was varied as indicated in the figure. The frequency of SAO was set to 0. Other parameters (d, *g*_3_, beta thalassaemia frequency) have no effect on the sensitivity of the test.

As shown in Fig3, even if the masking effect of alpha thalassaemia alone is relatively minor (*g*_1_* *=* *0·95; *g*_2_* *=* *0·75), an additional masking effect experienced only by those who inherit alpha thalassemia, heterozygous beta thalassaemia *and* G6PD deficiency could have a significant impact on the sensitivity of OTOFTs. Figure3 assumes that G6PD-deficient −α/−αββ^T^ individuals always test negative in a OTOFT, and illustrates the effect of varying *g*_4_: the probability of G6PD-deficient −α/ααββ^T^ individuals testing positive. In a population where the frequency of G6PD-deficient X chromosomes is 15% and the frequency of an ‘−α’ alpha thalassaemic deletion is 0·25 (both perfectly plausible in malaria-endemic regions), if *g*_4_ ≤0·4 then OTOFT sensitivity in males drops below 93%.

### A combination of alpha thalassaemia, G6PD deficiency and SAO could reduce the sensitivity of OTOFTs to <70%

**Figure 4:**
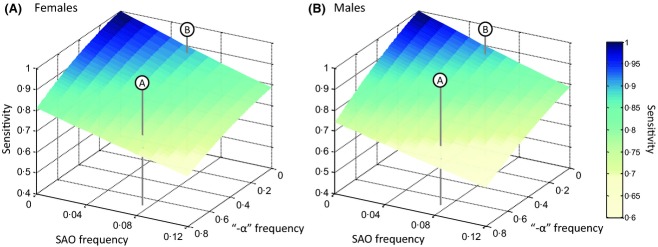
The combined impact of SAO, alpha thalassaemia frequency and G6PD deficiency on the sensitivity of one tube osmotic fragility tests for beta thalassaemia. The surfaces illustrate how the sensitivities of one tube osmotic fragility tests for beta thalassaemia change in males and females with changing frequencies of alpha thalassaemia (the ‘−α’ deletion) and the mutation responsible for Southeast Asian Ovalocytosis (SAO). *g*_1_* *=* *0·95, *g*_2_* *=* *0·75, *g*_4_ = 0·5, and *g*_5_ = 0. In both panels the population frequency of G6PD deficiency is 0.13. Marker ‘A’ indicates the maximum SAO and ‘−α’ frequencies that have been reported from the Northern coast of Papua New Guinea. Marker ‘B’ indicates the SAO frequency reported from Sumba Island, Indonesia, and a plausible Indonesian alpha thalassaemia frequency.

In Papua New Guinea, reported SAO frequencies range from 0·0005 to 0·074 (Patel *et al*, 2004); in Sumba Island, Indonesia, an SAO frequency of 0·057 has been reported (Shimizu *et al*, 2005). Even if SAO heterozygosity completely masks the osmotic fragility of beta thalassaemia carriers, such SAO frequencies alone will only reduce the sensitivity of OTOFTs as a test for beta thalassaemia to between 0·8 and 0·9 (Fig4).

However, populations in Papua New Guinea also carry some of the highest frequencies of alpha thalassaemia in the world, e.g. 0·68 in northern coastal populations (Flint *et al*, 1986). Furthermore, G6PD deficiency in populations in this region can reach 15% (Howes *et al*, 2012). If we assume that alpha thalassaemia has a small masking effect on the presence of heterozygous beta thalassaemia (*g*_1_* *=* *0·95, *g*_2_* *=* *0·75), but that this masking is exacerbated by the coinheritance of G6PD deficiency (*g*_4_ = 0·5, *g*_5_ = 0), the combined effect of alpha thalassaemia, SAO and G6PD deficiency is to reduce OTOFT sensitivity dramatically (Fig4). With an alpha thalassaemia frequency of 0·68; SAO frequency of 0·07, and G6PD deficient X chromosome frequency of 0.13, all entirely plausible for the Madang region of Papua New Guinea (Flint *et al*, 1986; Brabin & Brabin, 1990; Patel *et al*, 2004), the sensitivity of the OTOFT for beta thalassaemia drops to 0.74 in females and 0.69 in males (Fig4 marker A). Reported beta thalassaemia carrier frequencies in coastal Papua New Guinea reach up to 25% (Hill *et al*, 1988). The reported beta thalassaemia carrier frequency in Kar Kar Island, just a few kilometres off the coast from the high SAO/high alpha thalassaemia region of Madang, is 11·5%. Any attempt at OTOFT-based screening in these populations would be a matter of concern.

Certain regions of Indonesia may also present a problem due to the combination of SAO and alpha thalassaemia. Marker B in Fig4 pinpoints plausible population frequencies of SAO and alpha thalassaemia for Sumba Island, which has also been reported to possess a G6PD deficiency frequency of 8% (Shimizu *et al*, 2005). Although the sensitivity of the OTOFT predicted within our framework under these circumstances is higher, at 0.89 in females and 0.88 in males, it is still far from ideal. Furthermore, the alpha thalassaemia frequency we use for marker B (0·05), is consistent with the 11% and 10% carrier frequencies for alpha thalassaemia reported by Setianingsih *et al* (2003) in South Sulawesi and South Sumatera respectively, but alpha thalassaemia surveys specific to Sumba island are lacking. The true Sumba Island frequency of alpha thalassaemia may well be higher, given the history of high malaria transmission in the area.

### Alpha thalassaemia may have a more profound effect on the specificity of OTOFTs than iron deficiency

The specificity of a test is the reliability with which a positive test outcome indicates the presence of an underlying condition. In the case of beta thalassaemia, if a first-stage test is insufficiently specific, a great many samples from non-carriers may be sent for further testing. If medical budgets are limited, this may place an unacceptable strain on resources.

**Figure 5:**
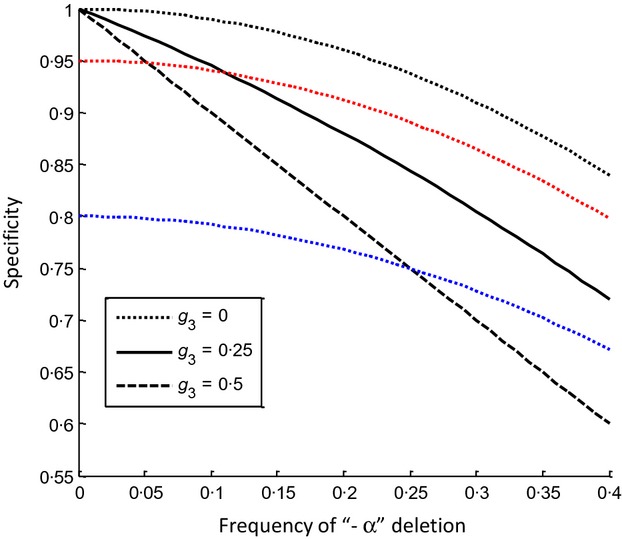
The effects of iron deficiency and alpha thalassaemia levels on the specificity of one tube osmotic fragility tests for beta thalassaemia. Parameter values were as follows: *g*_1_* *=* *1, *g*_2_* *=* *1; beta thalassaemia frequency = 0·12; SAO frequency = 0; G6PD frequency = 0. Different values of *g*_3_ are indicated by the different line styles: *g*_*3*_* *=* *0 (…); *g*_*3*_* *=* *0·25 (solid lines) and *g*_*3*_* *=* *0·5 (—). Different values of *d* are indicated by the different coloured lines: *d *=* *0 (black), *d *=* *0·05 (red) and *d *=* *0·2 (blue).

Regardless of epistasis between alpha and beta thalassaemia, or any effect of SAO or G6PD, the fact that the −α/−α ββ genotype leads to microcytosis means that there is likely to be a negative correlation between alpha thalassaemia frequency and OTOFT specificity for carriers of beta thalassaemia (Fig5). However, if heterozygosity for a single alpha thalassaemic deletion (−α/αα ββ) can *also* lead to microcytosis and a positive OTOFT, a probability represented here by the parameter *g*_3_, the relationship between alpha thalassaemia and OTOFT specificity declines more steeply as *g*_3_ becomes larger (Fig5).

A second factor that will affect the specificity of OTOFTs for carriers of beta thalassaemia is the level of iron deficiency in the population, represented by parameter *d*, the proportion of the population with a non-genetic cause of a positive OTOFT. Sumera *et al* (2012) showed that in a survey of 503 individuals, 174 were iron-deficient (determined by serum ferritin levels) and of those, 13% were OTOFT-positive. Using these values we might estimate *d *=* *0·05 (0·13 × 0·35). The study reported by Sumera *et al* (2012) was carried out in Pakistan, presumably on adults. A study of children in Egypt (El-Beshlawy *et al*, 2007) found that 310 out of 1000 individuals were iron-deficient (low serum iron, low transferrin saturation and normal to high total iron-binding capacity) and of those, 63·9% tested positive in a OTOFT. Based on these values, we might estimate *d *=* *0·2 (0·31 × 0·64). The red and blue dotted lines in Fig5 indicate the effect of these two values of *d* on the specificity of the test when *g*_3_* *= 0. For a screening programme carried out among the population in Pakistan studied by Sumera *et al* (2012), alpha thalassaemia is likely to cause greater losses in specificity than iron deficiency alone (red line) when *g*_3_ >0. In a population such as that studied by El-Beshlawy *et al* (2007)*,* iron deficiency alone already dramatically reduces the specificity of the test (blue line), but any additional effect of alpha thalassaemia will compound the problem.

## Discussion

Reliable methods of genetic screening are vital in regions where beta thalassaemia is common. A spate of recent studies (see Table III) have highlighted OTOFTs as sensitive, low-cost tests for beta thalassaemia heterozygosity, which can be carried out in virtually any setting. Despite these promising results, the analyses we present here caution against the large scale application of OTOFTs, due to the potential for epistasis between beta thalassaemia and other red blood cell variants to reduce the tests’ sensitivity.

All of the variants considered here: alpha thalassaemia, beta thalassaemia, G6PD deficiency and SAO, have malaria-protective properties (Williams, 2006), and therefore have distributions that are correlated with malaria exposure. If epistasis between these traits introduces any kind of systematic bias against accurate test results, such a problem is likely to be at its worst in areas with a history of high malaria exposure, where beta thalassaemia screening is most needed. Table III illustrates that the populations with the lowest OTOFT sensitivity are those with relatively high frequencies of beta thalassaemia.

Our analysis highlights important gaps in our understanding of OTOFTs. We introduced the parameters *g*_1_, *g*_2_ and *g*_3_, to represent respectively, the probabilities of −α/ααββ^T^ σσ; −α/−αββ^T^ σσ and −α/αα ββ σσ samples with normal G6PD activity testing positive, and *g*_4_ and *g*_5_ to take into account the possibility that G6PD deficiency may change the probability of −α/ααββ^T^ σσ and −α/−αββ^T^ σσ testing positive. As illustrated in Figs2, 3 and 5, selecting different values for these parameters has a huge effect on the predicted sensitivity and specificity of OTOFTs. However, as yet, we have no reliable estimates for these individual values, and how they change with the concentration of NaCl used in the test. Reliable estimates for *g*_1_ – *g*_5_, at different NaCl concentrations, and for different beta thalassaemic mutations would allow us to predict the likely specificity and sensitivity of OTOTFs with a higher degree of confidence, and may even make it possible to tailor the recommended NaCl concentration for the test according to the genetic background of the population. However, maintaining a very specific NaCl concentration in the field is already problematic.

To date, only one case report (Fucharoen *et al*, 2007) has studied the osmotic fragility profile of red blood cells from an individual heterozygous for both beta thalassaemia and SAO, demonstrating the potential for SAO to mask the osmotic resistance characteristic of beta thalassaemia carriers. Further study is clearly needed in this area. The model results we present here are arguably a worst-case scenario because it may not be that *all* beta thalassaemia heterozygotes who co-inherit SAO end up with a normal osmotic fragility profile. On the other hand, it is known that red blood cell membrane disorders in general have a tendency to increase the osmotic fragility of red blood cells (Da Costa *et al*, 2013). Other erythrocyte membrane abnormalities may, therefore, also counteract the osmotic resistance of cells from beta thalassaemia carriers. Although the variants responsible for other membrane abnormalities do not attain the same high frequencies as SAO in southeast Asia, their potential to hide carriers of beta thalassaemia may still be a cause for concern.

We considered one environmental factor: iron deficiency. However, we have not yet explored the possibility for other deficiencies, e.g. vitamin B12, to affect the sensitivity of OTOFTs by increasing the MCV of beta thalassaemia heterozygotes (Bilić *et al*, 2004). This would be an important addition to future work.

Other clinically important haemoglobinopathies - particularly sickle cell trait or haemoglobin C – exist alongside beta thalassaemia in many malarious or former malarious zones. Carriers of sickle cell trait or haemoglobin C have normal osmotic fragility, thus will be completely missed by screens that rely on OTOFTs alone as a first step. As reviewed by Giordano (2013), a truly effective screening programme anywhere in the world should now include a complete blood count, tests to examine red cell morphology, and either high performance liquid chromatography (HPLC) or capillary electrophoresis to separate and quantify the various haemoglobins in a sample. Such screening programmes would pick up carriers of beta thalassaemia without the need for OTOFTs.

However, the very reason OTOFTs are attractive is that tests such as those just described are expensive. Certain older electrophoretic or chromatographic methods to quantify HbA_2_ are reliable and much cheaper than modern HPLC (Weatherall & Clegg, 2001), so there may be a middle ground to be found, where accuracy is not sacrificed for the sake of cost. But, if absolutely the only economically viable option, are beta thalassaemia screens using OTOFTs still better than nothing? The studies summarized in Table III demonstrate that sensitivities of over 90% are possible in specific populations, and the authors of these studies have suggested OTOFTs have the potential to be useful tools in those settings. But if this approach is to be established elsewhere it will need prior assessment of the levels of at least alpha thalassaemia and G6PD deficiency in the population to be studied. Like all haemoglobin variants, these conditions occur at different frequencies in various parts of high frequency populations, and therefore micro-mapping of many centres is required. Unfortunately, the diagnosis of carriers of alpha thalassaemia (i.e. αα/−α; −α/−α or αα/− genotypes) can only be reliably accomplished by DNA analysis. In countries where facilities for this are not available they will have to form partnerships, either with rich countries (north/south partnerships) or with adjacent poorer countries where these techniques have been recently developed (south/south partnerships). The approaches to developing these partnerships has been clearly defined by the World Health Organization (WHO, 2002).

As we have shown here, in some populations, the sensitivity of the OTOFT for carriers of beta thalassaemia may drop below 70%. There must come a point at which a test is deemed too insensitive for widespread use. If nothing else, the results shown here are a strong argument for population-specific pilot studies in any regions where OTOFT-based screening is proposed. The health authorities in question should decide on an acceptable level of sensitivity before the pilot study begins.
